# Childhood growth associated with hip shapes at skeletal maturity: the Bergen Hip Cohort Study

**DOI:** 10.1186/s12891-025-09461-7

**Published:** 2025-12-30

**Authors:** Lene Bjerke Laborie, Francesco Sera, Kaya Kvarme Jacobsen, Trude Gundersen, Karen Rosendahl

**Affiliations:** 1 Mohn Medical Imaging and Visualization Centre, Department of Radiology, Haukeland University Hospital, Bergen, Norway; 2 Department of Clinical Medicine, University of Bergen, Bergen, Norway; 3 Department of Statistics, Computer Science and Applications G. Parenti, University of Florence, Florence, Italy; 4 Department of Orthopedic Surgery, District General Hospital of Førde, Førde, Norway; 5 Department of Orthopaedic Surgery, Haukeland University Hospital, Bergen, Norway; 6 Department of Radiology, University Hospital of North-Norway, Tromso, Norway; 7 Department of Clinical Medicine, UiT, The Arctic University of Norway, Tromso, Norway; 8 Department of Radiology, Pediatric Section, Haukeland University Hospital, Haukelandsbakken 15, Bergen, 5009 Norway

**Keywords:** Hip joint, Acetabulum, Growth

## Abstract

**Background and objectives:**

Abnormal joint shape is a known risk factor for osteoarthritis. We examined associations between growth trajectories during childhood, and acetabular shape at skeletal maturity.

**Methods:**

The prospective Bergen Hip Cohort Study provided anthropometric data on 1764 18-year-olds (59.0% female) with a median number of 10 measures of weight and BMI, and 11 measures of height, between birth and 12 years, and at follow-up age 18 years. At follow-up, four common radiological measurements characterising the acetabular shape, were measured on standardised hip radiographs. Growth trajectories were modelled using SuperImposition by Translation And Rotation (SITAR), separately for boys and girls, for weight, height and BMI, from birth until 18 years of age.

**Results:**

Six acetabular phenotypes were developed based on the four radiological measurements: Confirmed acetabular dysplasia (AD) was found in 3.4% (*n* = 61); a unilateral or bilateral tendency to AD in 15.9% (*n* = 280) and 5.4% (*n* = 96) respectively and unilateral or bilateral tendency to acetabular overcoverage in 15.4% (*n* = 271) and 8.3% (*n* = 146) and normal acetabular shape in 51.6% (*n* = 910). For males, bilateral tendency to acetabular overcoverage was associated with higher weight velocity in childhood [OR: 1.50; 95% CI: (1.15; 1.96), and bilateral tendency to acetabular overcoverage was associated with tempo of BMI in childhood. For females, no associations were observed with weight, but bilateral tendency to overcoverage was associated with higher height trajectories.

**Conclusions:**

Our analysis suggest that individual growth patterns in childhood are associated with modest variations in acetabular shape at skeletal maturity, especially in males.

**Clinical trial registry name and registration number:**

ClinicalTrialsGov NCT01818934, registered on 21th of March 2013. https://clinicaltrials.gov/.

**Supplementary Information:**

The online version contains supplementary material available at 10.1186/s12891-025-09461-7.

## Article summary

We examined associations between growth trajectories during childhood, And acetabular shape at skeletal maturity in the prospective, longitudinal Bergen Hip Cohort Study.

### What’s known on this subject

Abnormal acetabular shape is closely related to osteoarthritis predisposition of the hip joint.

### What this study adds

Individual growth patterns in childhood appear to be associated with modest variations in acetabular shape in young males

## Introduction

Osteoarthritis (OA) of the hip is a major public health problem world-wide, affecting around one in 10 of those aged 65 years and over [1]. Hip OA is the underlying cause in the majority of those requiring hip replacement over the age of 60, with hip developmental disorders present in a significant proportion in younger patients [2]. There has been increasing interest in the association of obesity and overweight with the subsequent onset and severity of hip OA and the requirement for hip joint replacement [3–5]. The mechanisms by which obesity contributes to the onset and progression of OA are not fully understood [6]. It has been hypothesised that the effects of obesity on the joint are predominantly due to increased biomechanical loading and its contribution to cartilage destruction, and a meta-analysis demonstrated a positive association between a high Body Mass Index (BMI) and the risk of OA [3]. Moreover, it has been suggested that metabolic factors associated with obesity may alter systemic levels of pro-inflammatory cytokines enhancing the production of proinflammatory factors in OA cartilage [6, 7].

Among the factors involved in the pathogenesis of hip OA, several features of hip joint architecture, such as acetabular dysplasia (AD) and femoroacetabular impingement (FAI), appear to play an important role, and may predate the development of OA by decades [2, 8, 9]. AD, or developmental dysplasia of the hip (DDH), is the most common musculoskeletal disorder in infancy, with a reported prevalence from 0.5% to 4.0% according to age, ethnicity and method of ascertainment [10]. It is a developmental disorder in which the hip joint forms incorrectly during fetal life and childhood. Compared with the normally shaped acetabulum, AD results in a smaller weight-bearing surface (i.e. undercoverage of the femoral head) leading to increased contact stress that might contribute to articular cartilage damage. FAI arises from one or more bony abnormalities that lead to abnormal contact between the acetabulum and the femoral head or neck. FAI can be categorized as cam-type or pincer-type: cam-type FAI results from a prominent head-neck junction, while pincer FAI results from a general or a localized acetabular overcoverage of the femur [11, 12]. Both impingement patterns cause articular cartilage and labral damage.

We hypothesized that obesity or rapid growth during infancy and childhood are associated with abnormal acetabular morphology at skeletal maturity as assessed by presence of AD (i.e. undercoverage of femoral head), or pincer-type FAI (i.e. overcoverage of the femoral head). We tested this hypothesis using data from the Bergen Hip Cohort Study. Data from this unique, prospective study affords an exceptional opportunity to examine the relation of weight and growth trajectories across the early life-course to radiological indices of acetabular morphology at skeletal maturity.

## Methods

### Study population

**Fig. 1:**
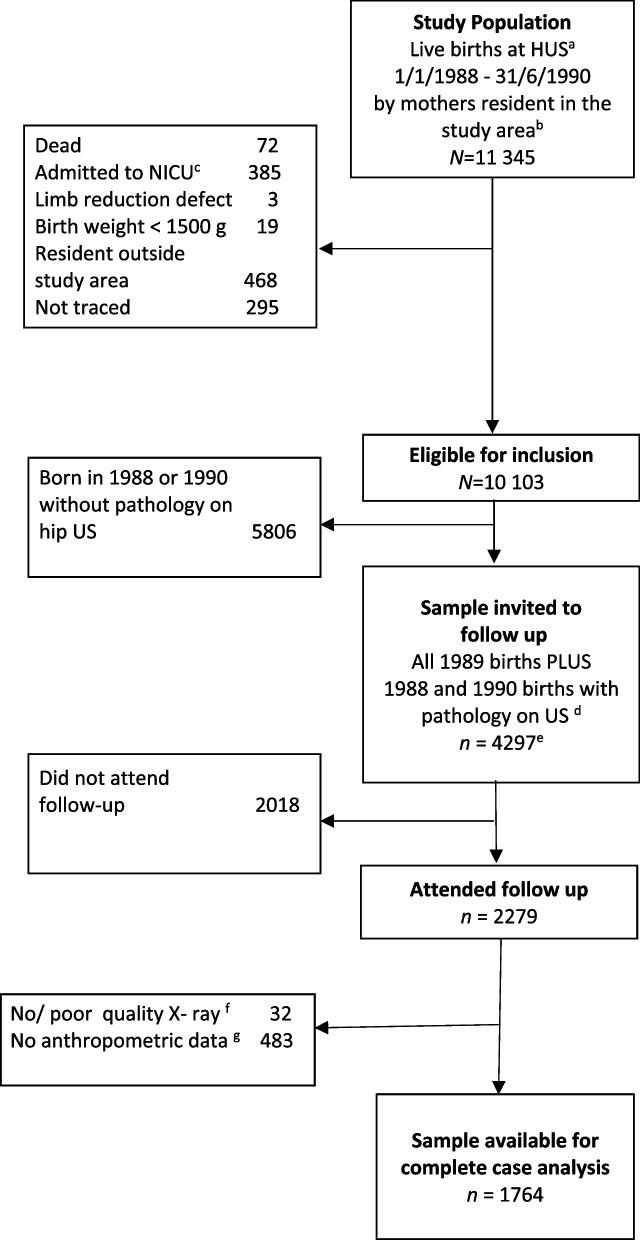
Sampling scheme of the Bergen Hip Cohort Study

The Bergen Hip Cohort Study is a prospective, longitudinal study following participants in a randomised clinical trial of newborn screening to adult life, and which includes standardised ultrasound and radiographic examinations of the hip in the newborn period and at skeletal maturity respectively. All infants (*n* = 11 925) born alive at Haukeland University Hospital (HUS) in Bergen between 1 st January 1988 and 30th June 1990 were enrolled in a randomized clinical trial (RCT) to evaluate the effect of three different screening strategies for DDH. Further details of recruitment and baseline characteristics to the RCT are described elsewhere [13, 14]. The Bergen Hip Cohort Study comprised 11 345 infants (Fig. 1). At the time of follow up, between 1 st February 2007 and 1 st April 2009, 4297 trial participants were invited to attend a clinical and radiological examination, of whom 2 279 (53.0%) attended, and of whom 1764 were available for the final analysis (Fig. 1).

### Clinical and radiological examinations

**Fig. 2:**
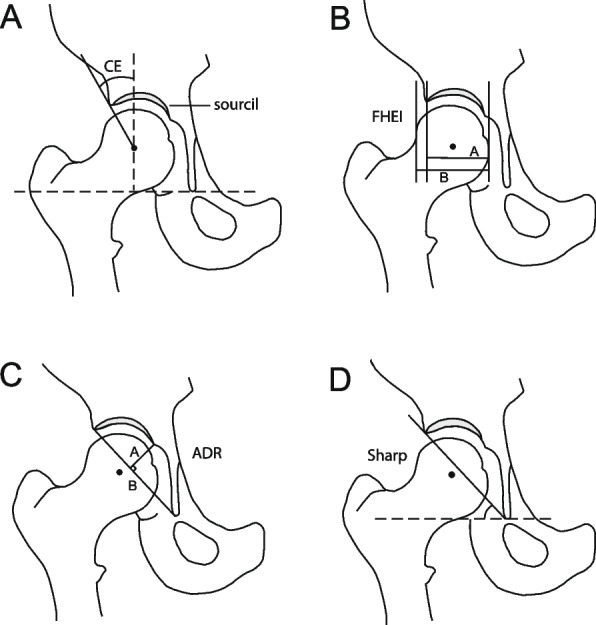
Radiological markers for hip dysplasia. **a** The Centre-Edge (CE) angle of Wiberg: The angle between a vertical line through the centre of the femoral head and perpendicular to the horizontal tear drop line, and a line from the centre of the femoral head extending to the lateral edge of the acetabulum. **b** The Femoral Head Extrusion Index: A ratio between the amount of the femoral head covered by the acetabulum (A), and the total width of the femoral head (B). (A/B × 100). **c** The Acetabular Depth Ratio (ADR): The depth of the acetabulum (A), divided by the width of the acetabulum (B), and multiplied by 1000 (A/B × 1000). The depth is measured perpendicularly from the midpoint of line (B). The width is measured between the lateral rim of the acetabulum, to the inferior end of the tear drop. **d** Sharp’s angle: The angle between a line from the tip of the pelvic tear drop to the lateral margin of the acetabulum, and a horizontal line through the tip of the pelvic tear drop

At follow up, all participants underwent a standardised clinical and radiological examination (Suppl.info document), which included measurement of body weight and standing height. All radiological examinations were performed at the Department of Radiology, Haukeland University Hospital, by a single, specially trained radiographer using a low-dose Digital Radiography technique (DigitalDiagnost System, version 1·5, Philips Medical Systems, Hamburg, Germany). For the present analysis, we used the weight bearing anteroposterior (AP) pelvic radiograph, taken with feet pointing forward to align femurs according to the vertical parallel axis, and a film-focus distance of 1·2 m centred 2 cm above the pubic symphysis. All radiographs were measured by one of three project members, masked to the clinical or weight status of the participant using a validated digital measurement program (University of Iowa Hospitals and Clinics, Iowa City, Iowa, USA) [15, 16]. For the radiological measurements, a detailed calibration process was carried out and the inter- and intra-observer variability demonstrated only minor differences, as reported previously [15]. Four parameters assessing acetabular shape were measured in each hip: Sharp’s angle, Wiberg’s centre-edge (CE) angle, Heyman and Hendon’s femoral head extrusion index (FHEI), and the acetabular depth-width ratio (ADR) (Fig. 2), with distributions previously reported for the follow-up participants [17].

### Primary outcome

**Fig. 3:**
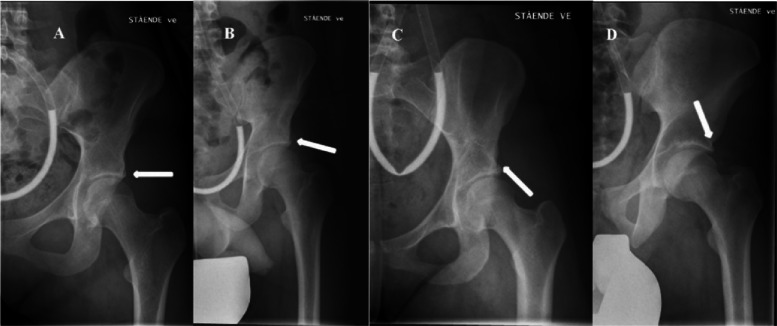
Radiographs of four participants with acetabular phenotypes classified according to latent class method. In the normal phenotype (**A**), the weight-bearing, bony area of the acetabulum, seen as a hyper-dense line along the lateral part of the acetabular roof, curves slightly downwards. This provides optimal coverage and support for the femoral head, without interfering with the femoral head-neck junction during movement. In hips with a tendency to AD (**B**) or with AD (**C**), the lateral part of the acetabulum has a more horizontal, or upward direction. In hips with over-coverage, also known as pincer-type FAI (**D**), the lateral part of the acetabulum has a more pronounced downward curve. This lateral extension can cause the acetabulum to interfer with the femoral head-neck junction during movement

We developed acetabular shape phenotypes based on the four radiology parameters described above. These parameters were categorised using cut-off values (Supplementary Table S1) and then combined using latent class analysis. Latent class analyses were performed on the right and left side separately using poLCA, an R package for polytomous variable latent class analysis [18]. Models with one to ten classes were estimated, and the final model with four classes was chosen based on the smallest value of the Bayesian Information Criterion. For each participant, each hip was assigned to their most likely class based on posterior probabilities, and classes were given subjective labels based on the distribution of the radiological marker among the four latent classes (Supplementary Table S2). For both hips the four classes were defined as Normal; Tendency to Acetabular dysplasia; Acetabular dysplasia; Acetabular overcoverage (Fig. 3). Finally, each participant was assigned to one of the six following mutually exclusive categories created by combining the information for both hips: Normal; Unilateral tendency to Acetabular Dysplasia: Bilateral tendency to Acetabular Dysplasia; confirmed Acetabular Dysplasia in one or both hips; Unilateral tendency to Acetabular overcoverage; Bilateral tendency to Acetabular overcoverage. (Supplementary Table S3 and S4). These six categories comprised the primary outcome measure.

### Main exposure variable

In Norway, body weight and height are regularly measured at child health clinics and entered on paper records as part of routine community child health services. Weight is measured at six weeks of age, at four, five, six, 10, 11 and 18 months, and at two, four, six, eight and 12 years. Length is measured at six and 18 months, and height at two, five, six and 12 years. We retrieved the paper records for all consenting participants from one of 26 Bergen city clinics and entered them on a database. Initial data inspection was undertaken to identify errors in date of measurement and/or data entry. Standard deviations (SD) were calculated for each measure at each age and values greater than or lower than 5 SD from the mean excluded from analysis. After excluding measurement or recording errors and extreme values, 19 783 records were available for 1764 participants, representing a median number of 10 measures of weight, and 11 measures of height during childhood per participant. Based on the weight and height measurements, Body Mass Index (BMI) was calculated for a total of 10 measurements per patient.

### Derivation of growth parameters

The SITAR (SuperImposition by Translation And Rotation) model was used to reduce the dimensionality of the anthropometric data and to summarise individual growth curves [19]. SITAR is a form of mixed effects growth curve analysis that models on both measurement and age scales to describe how each individual differs from the mean curve, representing three different parameters of growth: subject’s size, growth tempo and growth velocity. The subject’s size is reflected in up/down shift from the mean curve. The growth tempo is reflected in left/right shift (on the age scale) which corresponds to the relative timing of adiposity rebound, which represents the point in a child's development where their BMI starts to increase again following a period of decline during early childhood. After an initial period of decreasing BMI during the first few years of life, this second increase in BMI is a normal part of growth and development, but the timing of the rebound is considered an indicator of future obesity risk [20]. The velocity is reflected in stretching/shrinking of the age scale and hence describing differences in the growth rate. SITAR models were fitted separately for boys and girls to model weight, height and BMI trajectories until 18 years of age. In all models, age and weight, height and BMI were initially log-transformed. For the analysis of height and weight trajectories we considered models with three knots spaced equally across age and we parametrised size and velocity as random growth parameters. For the analysis of BMI trajectories, we considered models with four knots spaced equally across age, with size, tempo and velocity as random growth parameters. The age scale was centred at six years.

### Birth registration records

Child and maternal characteristics, including labour and delivery details, were extracted for all participants from birth records held in the national Norwegian Medical Birth Registry. The following variables were extracted: date of birth; mode of delivery (caesarean section, forceps); breech presentation at delivery; weight and length at birth; transfer to neonatal intensive care; presence of limb reduction defects; indication for abduction treatment (Frejka pillow) for neonatal hip dysplasia.

### Statistical methods

The main determinants of attendance at follow up were found to be sex, indication for abduction treatment, and history of newborn ultrasound examination, whereas breach position, caesarean section, forceps use, birth weight, and randomization group during the initial RCT did not affect attendance (see Supplementary Table S5). We used inverse probability-weights to take account of the study sampling design and non-response [21]. Weights were calculated as the inverse of the product of the sampling probability (function of birth year and positive results of newborn ultrasound examination), and the attendance to follow up probability was predicted from a logistic model with sex, indication for abduction treatment, and history of newborn ultrasound examination as predictors, as these factors were found to be the main determinants of attendance at follow up. We calculated descriptive statistics, namely proportions for categorical variables and mean and standard deviation for quantitative variables.

Univariate associations between acetabular phenotypes and explanatory variables were estimated using weighted polytomous logistic regression with acetabular phenotype as outcome, and each variable as exposures. For each anthropometric variable measured at follow up (weight, height, BMI), differences (with 95% confidence intervals) among acetabular phenotypes with respect to the ‘normal’ phenotype group were assessed using weighted regression models.

Associations between acetabular phenotypes and SITAR growth random effects parameters (size, tempo and velocity) were estimated using multivariable weighted polytomous logistic regression with hip phenotype as outcome, and the derived growth random effect parameters as exposures for boys and girls separately. The models were mutually adjusted for the other growth random effect parameters and for the following covariates (breech position at delivery, family history of acetabular dysplasia, and previous abduction treatment).

### Ethics

All participants gave written informed consent according to the 1964 Declaration of Helsinki at time of follow-up in 2007–09. All participants were above age 16 years at time of follow-up and at time of data collection in 2007–09, and informed consent from parents or legal guardians was therefore not required nor obtained at time of follow-up. Informed consent to participate was obtained from the parents or legal guardians for all participants in the neonatal period for the initial RCT in 1988–90. The study research protocol, including retrospective data collection and analyses of the non-responders, was approved by the Medical Research Ethics Committee of the Western region of Norway (No. 20594).

## Results

The characteristics of those attending for follow up (*n* = 2279; 60.9% women) and those with complete data (*n* = 1764; 59.0% women) were comparable with respect to perinatal, demographic and anthropometric variables measured at enrolment (Table 1). Young women were over-represented at follow-up (60.9%), reflecting the sampling design and their higher propensity to participate in the follow up examination. Among those who attended follow up, mean age was 18.6 years, 10.2% were born by caesarean section, 5.1% were in a breech position at birth, 9.3% received abduction treatment for neonatal hip dysplasia, and a family history of hip disorders was self-reported in the questionnaire by 11.4%. Descriptives for the subjects with anthropometric data (*n* = 1764) were similar to the descriptives of all the participants that attended to the follow up (Table 1).

**Table 1:** Baseline Characteristics of the Study Population. Of the 2279 young adults who attended at follow-up, 1764 had complete radiological and anthropometric data

Variable	Attended at follow up (*N* = 2279)		Complete cases (*N* = 1764)	
	n	%	n	%
Sex				
Male	890	39.1	724	41.0
Female	1,389	60.9	1,040	59.0
Year of Birth				
1988	182	8.0	92	5.2
1989	1,984	87.1	1,611	91.3
1990	113	5.0	61	3.5
Breech position				
No	2,163	94.9	1,668	94.6
Yes	116	5.1	96	5.4
Caesarean section				
No	2,046	89.8	1,576	89.3
Yes	233	10.2	188	10.7
Forceps				
No	2,189	96.1	1,689	95.8
Yes	90	3.9	75	4.3
Randomisation group				
General screening	766	33.6	567	32.1
Selective screening	719	31.5	561	31.8
Clinical screening	794	34.8	636	36.1
Newborn Ultrasound				
No	1,323	58.1	1,062	60.2
Yes	956	41.9	702	39.8
Frejka Pillow treatment				
No	2,066	90.7	1,634	92.6
Yes	213	9.3	130	7.4
Family History of Hip Disorders				
No	2,020	88.6	1,587	90.0
Yes	259	11.4	177	10.0
Birth weight (g)	Mean	SD	Mean	SD
Male	3,634.1	530.7	3,632.2	531.5
Female	3,500.8	484.2	3,500.3	475.0
Age at follow up (years)	18.6	0.5	18.6	0.5

The acetabular phenotype was considered normal in just over half of the participants (*n* = 910) corresponding to a weighted prevalence of 52%. Confirmed AD was found in 3.4% (*n* = 61), and a unilateral or bilateral tendency to AD in 15.9% (*n* = 280) and 5.4% (*n* = 96) respectively (Table 2). Tendency to Acetabular overcoverage phenotype was observed in one fifth of the sample (23.9%; *n* = 417), of which 15.4% (*n* = 271) were uni- and 8.3% (*n* = 146) were bilateral. These phenotypes differed with respect to demographic, perinatal, and clinical findings. Unilateral acetabular overcoverage was more prevalent in females (18.4%) than boys (11.9%) [Odds Ratio (OR): 1.70; 95% Confidence Interval (CI): 1.27; 2.28]. A bilateral tendency to AD was more prevalent in those with a breech presentation at birth (12.3%) than those with a cephalic presentation (5.1%) [OR: 2.53; 95% CI: 1.19; 5.37]. Confirmed AD was more prevalent in those previously treated with an abduction device (9.5% vs 3.1%; [OR: 3.33; 95% CI 1.54; 7.17], and those with a family history of hip disorders (7.3% vs 2.9%; [OR 2.64; 95% CI 1.28; 5.42]. There were otherwise no differences with respect to newborn screening randomisation group, family history, or prevalence of reported hip pain or discomfort, joint laxity, or physical activity at follow up.

**Table 2:** Association of covariates with the different hip phenotypes for the 1764 participants. Each row represents a covariate and sums up to 100%

	**Normal (*n* = 910)**	**Unilateral Tendency to AD (*n* = 280)**			**Bilateral Tendency to AD (*n* = 96)**			**Confirmed AD (*n* = 61)**			**Unilateral Acetabular Overcoverage (*n* = 271)**			**Bilateral Acetabular Overcoverage (*n* = 146)**		
Prevalence (%)*	51.6	15.9			5.4			3.4			15.4			8.3		
**Variable**	**%**	**%**	**OR**	**95% CI**	**%**	**OR**	**95% CI**	**%**	**OR**	**95% CI**	**%**	**OR**	**95% CI**	**%**	**OR**	**95% CI**
**Sex**																
Male	54.4	14.8			6.0			3.5			11.9			9.4		
Female	49.4	15.8	1.18	(0.89; 1.56)	4.8	0.89	(0.58; 1.37)	3.2	1.01	(0.58; 1.73)	**18.4**	**1.70**	**(1.27; 2.28)**	8.3	0.98	(0.68; 1.40)
**Breech position at birth**																
No	52.1	15.1			5.1			3.3			15.2			8.1		
Yes	49.7	18.5	1.28	(0.69; 2.57)	12.3	**2.53**	**(1.19; 5.37)**	3.8	1.21	(0.44; 3.34)	11.8	0.81	(0.37; 1.78)	3.9	0.45	(0.15; 1.38)
**Family history of hip disorders**																
No	52.3	15.3			5.2			2.9			15.3			8.9		
Yes	49.3	14.7	1.01	(0.62; 1.65)	7.9	1.60	(0.83; 3.07)	**7.3**	**2.64**	**(1.28; 5.42)**	12.0	0.83	(0.90; 1.41)	8.4	0.99	(0.52; 1.88)
**Pillow treatment**																
No	52.1	15.3			5.3			3.1			15.3			8.9		
Yes	47.6	15.6	1.12	(0.66; 1.89)	9.5	1.96	(0.91; 4.20)	9.5	**3.33**	**(1.54; 7.17)**	10.2	0.73	(0.40; 1.35)	7.6	0.94	(0.41; 2.16)
**Hip pain or discomfort**																
None	52.5	14.7			5.7			3.5			14.5			9.0		
Moderate/strong	48.7	18.0	1.32	(0.92; 1.90)	4.2	0.80	(0.42; 1.52)	2.6	0.80	(0.37; 1.76)	18.2	1.35	(0.94; 1.94)	8.2	0.99	(0.63, 1.61)
**Beighton hypermobility score > = 4**																
No	52.1	14.9			5.4			3.2			15.3			9.0		
Yes	50.8	18.6	1.29	(0.86; 1.93)	6.0	1.14	(0.61; 2.15)	4.3	1.35	(0.64; 2.86)	12.8	0.86	(0.55; 1.33)	7.5	0.86	(0.48; 1.52)
**Weekly physical activity (hours/week)**																
None	50.0	14.4			5.7			2.4			18.1			9.4		
< 1 h	56.7	14.4	0.88	(0.60; 1.29)	3.1	0.48	(0.25; 0.92)	3.1	1.13	(0.50; 3.53)	13.0	**0.63**	**(0.43; 0.92)**	9.7	0.90	(0.57; 1.44)
2–6 h	52.3	13.5	0.89	(0.59; 1.35)	8.2	1.37	(0.70; 1.24)	4.7	1.86	(0.86; 4.01)	13.4	0.70	(0.48; 1.04)	8.0	0.82	(0.49; 1.37)
> 7 h	48.4	21.0	1.51	(1.00; 2.29)	4.7	0.86	(0.43; 1.73)	3.9	1.66	(0.71; 3.88)	15.7	0.83	(0.59; 1.36)	6.3	0.70	(0.39; 1.26)
**Randomisation group**																
General screening	48.5	16.3			6.5			3.6			16.7			8.5		
Selective screening	53.2	15.5	0.87	(0.61; 1.24)	4.7	0.66	(0.38; 1.17)	3.6	0.90	(0.46; 1.78)	14.4	0.79	(0.55; 1.13)	8.6	0.92	(0.58; 1.48)
Clinical screening	53.6	14.4	0.80	(0.57; 1.13)	5.3	0.74	(0.44; 1.26)	3.0	0.74	(0.37; 1.49)	13.3	0.78	(0.55; 1.10)	9.4	1.00	(0.64; 1.57)
**Newborn Ultrasound**																
No	53.9	14.5			5.2			3.2			14.1			9.1		
Yes	48.6	16.8	1.29	(0.96; 1.72)	5.9	1.25	(0.79; 1.98)	3.6	1.22	(0.69; 2.15)	16.7	1.31	(0.98; 1.76)	8.5	1.04	(0.71; 1.52)

^*^weighted prevalence

At follow up, young men with bilateral tendency to acetabular overcoverage were on average 5.7 kg [95% CI 0.7; 10.6 kg] heavier and had a BMI 1.3 kg/m^2^ [95% CI 0.04; 1.3 kg/m^2^] greater than those with a normal hip phenotype. Young men with uni- or bilateral tendency to acetabular overcoverage were both more likely to be categorised as obese (Table 3). Young women with a uni- or bilateral tendency to acetabular overcoverage were on average respectively 1.5 cm [95% CI 0.5; 2.6 cm] and 2.1 cm [95% CI 0.7; 3.5 cm] taller than those with a normal hip phenotype but did not otherwise demonstrate differences in anthropometric measures by hip phenotype.

**Table 3:** The distribution of mean weight, height, BMI, overweight and obese categories at follow up examination, by sex and hip phenotypes

	**Males**			**Difference**	**Females**			
	**n**	**Mean**	**SE**	**95% CI**	**n**	**Mean**	**SE**	**95% CI**
**Weight (kg)**								
*All*	*718*	*75.7*	*0.5*		*1032*	*63.4*	*0.4*	
Normal	395	75.0	0.6	Ref	509	63.0	0.5	Ref
Unilateral tendency to AD	104	74.1	1.1	−0.9 (−3.4; 1.6)	171	63.1	0.9	0.1 (−1.9; 2.0)
Bilateral tendency to AD	43	76.9	2.1	1.9 (−2.4; 6.1)	53	61.3	1.3	−1.7 (−4.4; 1.0)
Confirmed AD	26	75.5	1.9	0.5 (−3.3; 4.4)	35	65.4	2.4	2.4 (−2.4; 7.3)
Unilateral tendency to overcoverage	86	76.3	1.7	1.3 (−2.2; 4.8)	184	64.2	1.0	1.2 (−1.1; 3.9)
Bilateral tendency to overcoverage	64	**80.7**	**2.4**	**5.7 (0.7; 10.6)**	80	65.0	1.4	2.1 (−0.8; 4.9)
**Height (cm)**								
*All*	*719*	*180.3*	*0.3*		*1033*	*166.6*	*0.2*	
Normal	395	180.4	0.3	Ref	*510*	166.2	0.3	Ref
Unilateral tendency to AD	104	179.8	0.6	−0.6 (−2.0; 0.8)	*171*	166.1	0.4	−0.1 (−1.1; 0.9)
Bilateral tendency to AD	43	180.3	1.1	−0.1 (−2.3; 2.1)	*53*	164.6	0.7	−1.6 (−3.1; 0.03)
Confirmed AD	26	179.9	1.6	−0.5 (−3.8; 2.8)	*35*	166.8	0.8	0.7 (−1.6; 2.9)
Unilateral tendency to overcoverage	86	179.5	0.7	−0.9 (−2.5; 0.7)	184	**167.7**	**0.5**	**1.5 (0.5; 2.6)**
Bilateral tendency to overcoverage	64	181.7	0.7	1.3 (−0.2; 2.9)	80	**168.3**	**0.7**	**2.1 (0.7; 3.5)**
**BMI (kg/m**^**2**^**)**								
*All*	*718*	*23.2*	*0.1*		*1031*	*22.8*	*0.1*	
Normal	*395*	23.0	0.2	Ref	*509*	22.8	0.2	Ref
Unilateral tendency to AD	*104*	22.9	0.3	−0.1 (−0.9; 0.6)	*170*	22.9	0.3	0.1 (−0.6; 0.8)
Bilateral tendency to AD	*43*	23.6	0.5	0.6 (−0.6; 1.7)	*53*	22.6	0.4	−0.2 (−1.1; 0.7)
Confirmed AD	*26*	23.3	0.4	0.3 (−0.7; 1.2)	*35*	23.4	0.7	0.7 (−0.8; 2.7)
Unilateral tendency to overcoverage	86	23.6	0.5	0.9 (0.04; 1.3)	*184*	22.8	0.3	−0.02 (−0.7; 0.7)
Bilateral tendency to overcoverage	64	24.4	0.7	1.3 (−0.1; 2.8)	*80*	23.0	0.5	0.2 (−0.8; 1.2)
**Obese (BMI > = 30)**	**n**	**Prevalence**	**SE**	**OR 95% CI**	**n**	**Prevalence**	**SE**	**p value**
*All*	*718*	*6.5*	*0.9*		*1031*	*5.5*	*0.7*	
Normal	*395*	5.1	1.1	Ref	*509*	4.7	1.0	Ref
Unilateral tendency to AD	*104*	4.0	2.0	0.8 (0.3; 2.4)	*170*	7.6	2.1	1.6 (0.7; 3.3)
Bilateral tendency to AD	*43*	3.6	2.6	0.7 (0.1; 3.4)	*53*	3.0	2.3	0.6 (0.1; 2.9)
Confirmed AD	*26*	0	-	-	*35*	8.4	4.8	2.0 (0.5; 7.2)
Unilateral tendency to overcoverage	86	11.1	3.5	2.3 (1.0; 5.2)	*184*	6.1	1.8	1.2 (0.6; 2.6)
Bilateral tendency to overcoverage	64	**16.9**	**4.7**	**3.5 (1.6; 7.9)**	*80*	5.6	2.6	1.2 (0.4; 3.5)
**Overweight (BMI [25,30))**								
*All*	*718*	*18.1*	*1.4*		*1031*	*15.5*	*1.2*	
Normal	*395*	18.6	2.0	Ref	*509*	18.1	1.7	Ref
Unilateral tendency to AD	*104*	19.9	4.0	1.1 (0.6; 1.9)	*170*	11.0	2.4	0.6 (0.3; 1.0)
Bilateral tendency to AD	*43*	19.4	6.2	1.0 (0.5; 2.3)	*53*	11.1	4.4	0.5 (0.2; 1.4)
Confirmed AD	*26*	21.8	8.1	1.1 (0.5; 1.4)	*35*	20.3	6.9	1.2 (0.5; 2.9)
Unilateral tendency to overcoverage	86	16.2	4.0	0.9 (0.5; 1.7)	*184*	11.9	2.5	0.6 (0.5; 1.0)
Bilateral tendency to overcoverage	64	12.8	4.4	0.7 (0.3; 1.7)	*80*	17.8	4.1	1.0 (0.5; 1.9)

**Fig. 4:**
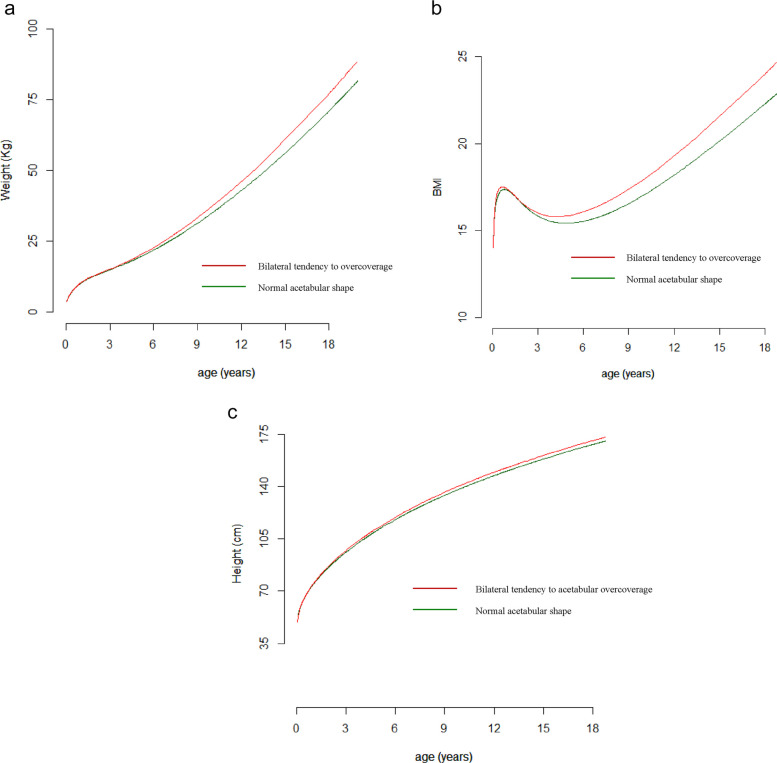
**a** Boys, mean predicted weight (Kg) trajectory between birth to 18 years of age by bilateral tendency to acetabular overcoverage (red line) and Normal (green line) acetabular shape group. **b** Boys, mean predicted BMI trajectory between birth to 18 years of age by bilateral tendency to acetabular overcoverage (red line) and normal (green line) acetabular shape. **c** Girls, mean predicted height trajectory between birth to 18 years of age by bilateral tendency to acetabular overcoverage (red line) and normal (green line) acetabular shape

For males, bilateral tendency to acetabular overcoverage was associated with higher weight velocity in childhood [OR: 1.50; 95% CI 1.15; 1.96] for each unit standard deviation increase (Table 4) (Fig. 4 a). For males, bilateral tendency to acetabular overcoverage was associated with tempo of BMI in childhood [OR: 0.67; 95% CI 0.45; 0.95 (Fig. 4 b). These results indicate that young men with a bilateral tendency to acetabular overcoverage have lower values of the tempo-BMI parameter, with lower values representing anticipated tempo compared with the average tempo (a leftward shift or translation of the BMI velocity curve). Consequently, on average young men with a bilateral tendency to acetabular overcoverage had a higher early life BMI peak followed by an earlier adiposity rebound with a consequent higher velocity until skeletal maturity relative to those with a normal acetabular phenotype.

**Table 4:** Results of multivariable analyses of associations between standardised random effects (size, tempo and velocity) estimated by SITAR models and acetabular phenotype assessed at 18 years for males (*n* = 724) and females (*n* = 1040) separately

	**Unilateral tendency to AD**		**Bilateral tendency to AD**		**Confirmed AD**		**Unilateral tendency to overcoverage**		**Bilateral tendency to overcoverage**	
	**OR**	**95% CI**	**OR**	**95% CI**	**OR**	**95% CI**	**OR**	**95% CI**	**OR**	**95% CI**
**Females (*n* = 1040)**										
**Weight (*n* = 871)**										
Size	1.07	(0.88; 1.30)	0.79	(0.55; 1.14)	1.13	(0.79; 1.64)	1.06	(0.87; 1.29)	1.22	(0.92; 1.63)
Velocity	1.11	(0.90; 1.38)	1.09	(0.81; 1.47)	1.05	(0.65; 1.71)	1.21	(0.99; 1.48)	0.90	(0.99; 1.49)
**Length/height (*n* = 898)**										
Size	1.05	(0.86; 1.27)	0.74	(0.50; 1.10)	1.21	(0.81; 1.78)	1.11	(0.92; 1.35)	1.08	(0.83; 1.40)
Velocity	1.06	(0.87; 1.30)	1.33	(0.87; 2.01)	0.77	(0.52; 1.13)	1.22	(0.59; 1.49)	**1.42**	**(1.09; 1.86)**
**BMI (*n* = 845)**										
Size	1.04	(0.83; 1.30)	0.80	(0.53; 1.19)	1.15	(0.77; 1.73)	0.95	(0.76; 1.19)	1.25	(0.91; 1.72)
Tempo	0.95	(0.76; 1.20)	**1.36**	**(1.03; 1.79)**	0.78	(0.56; 1.11)	1.02	(0.82; 1.27)	1.02	(0.76; 1.37)
Velocity	1.04	(0.81; 1.34)	1.35	(0.93; 1.96)	0.98	(0.60; 1.61)	1.08	(0.86; 1.37)	0.91	(0.68; 1.21)
**Males (*n* = 724)**										
**Weight (*n* = 601)**										
Size	0.85	(0.65; 1.10)	1.13	(0.80; 1.61)	1.45	(0.89; 2.36)	0.92	(0.72; 1.18)	1.08	(0.81; 1.43)
Velocity	1.06	(0.62; 1.38)	1.16	(0.80; 1.68)	1.01	(0.70; 1.47)	0.94	(0.68; 1.30)	**1.50**	**(1.15; 1.96)**
**Length/height (*n* = 632)**										
Size	0.93	(0.73; 1.18)	1.04	(0.73; 1.47)	1.20	(0.76; 1.88)	0.94	(0.74; 1.22)	1.02	(0.78; 1.34)
Velocity	0.88	(0.67; 1.12)	1.09	(0.70; 1.68)	0.84	(0.49; 144)	0.85	(0.65; 1.13)	1.16	(0.90; 1.49)
**BMI (*n* = 583)**										
Size	0.81	(0.59; 1.10)	1.42	(0.88; 2.28)	1.51	(0.93; 2.46)	0.97	(0.74; 1.28)	1.09	(0.82; 1.48)
Tempo	0.80	(0.58; 1.09)	0.67	(0.44; 1.01)	0.98	(0.64; 1.50)	0.92	(0.67; 1.27)	**0.67**	**(0.45; 0.95)**
Velocity	0.90	(0.65; 1.25)	0.63	(0.36; 1.09)	0.94	(0.56; 1.60)	0.90	(0.69; 1.19)	1.13	(0.74; 1.72)

Young women with a bilateral tendency to acetabular overcoverage were characterized by higher velocity of childhood height trajectories [OR: 1.42; 95% CI: 1.09; 1.86] (Fig. 4 d). For young women with a bilateral tendency to acetabular dysplasia there was an association with tempo of BMI in childhood [OR: 1.36; 95% CI: 1.03; 1.79], with a BMI trajectory characterised by later BMI peak velocity and delayed adiposity rebound. No associations were observed with weight in young women.

## Discussion

This prospective, longitudinal study suggests an association between growth patterns during infancy and childhood, and acetabular shape as assessed on radiographs at skeletal maturity.

For males, bilateral tendency to acetabular overcoverage was associated with higher weight velocity in childhood. Furthermore, bilateral tendency to acetabular overcoverage were associated with tempo of BMI in childhood. On average, young men with a bilateral tendency to acetabular overcoverage had a higher early life BMI peak followed by an earlier adiposity rebound with a consequent higher velocity until skeletal maturity relative to those with a normal hip phenotype. For females, no associations were observed with weight. We noticed however an association with height trajectories, in that females with a bilateral tendency to acetabular overcoverage were characterized by higher velocity of childhood height trajectories. For young women with a bilateral tendency to acetabular dysplasia there was an association with tempo of BMI in childhood [OR: 1.36; 95% CI: 1.03; 1.79], with a BMI trajectory characterised by later BMI peak velocity and delayed adiposity rebound.

Our observational study suggests that specific acetabular phenotypes, particularly overcoverage, are associated with distinct growth trajectories from birth to adulthood. In particular, overcoverage in skeletally mature males appears to be associated to higher weight and obesity in childhood. These findings suggest a potential role of systemic growth factors beyond traditional mechanical theories impacting the acetabular morphology. The study was, however, not designed to scrutinize causal effects. Genetic and hormonal/growth factors might influence both systemic skeletal growth and skeletal morphology, and as such might impact both growth patterns during infancy and childhood and acetabular shape at skeletal maturity [22, 23]. Biomechanical factors significantly impact acetabular shape, but further research is required to investigate if acetabular morphology also affects systemic growth in childhood [24–28]. Further research is also needed to investigate the clinical significance of the associations between childhood growth patterns, in particular obesity, and acetabular shape at skeletal maturity.

Previous studies, using statistical shape modelling (SSM) to identify subtle shape variations, have indicated associations between the shape of the proximal femur and radiographic osteoarthritis in middle-aged and elderly individuals, based on DXA images at baseline and after 5–6 years [24, 29, 30]. Others have used SSM based on DXA images from 14- and 18-year-olds and serial height measurements collected between age 5–20 years [31]. Their findings, using SITAR mixed effects growth curve analysis, indicate that height tempo (corresponding to pubertal timing) at age 14 was associated with hip shape modes which may be related to future risk of hip OA and/or fracture [31]. There was little relationship between tempo and proximal femur shape at age 18. A similarly designed study from Staines and colleagues, based on SSM from DXA scans of 60–64-year-olds and height data collected at ages 2–15 years, suggested that individual growth patterns, particularly in the adolescent period were associated with variations in hip shape at 60–64 years, which are consistent with features seen in OA [32]. To sum up these studies, although many potential confounders were controlled for, the results are heterogenous, reflecting differences between the cohorts as well as differences in positioning of the patients, imaging techniques and equipment used. Another issue is the use of DXA-images to assess hip morphology. Although modern scanners using narrow-angle fan-beam technology has improved image quality, spatial resolution, being crucial for identifying reference points in an image, remains inferior to conventional radiography [24, 33]. A recent study on 411 individuals, comparing automated measurements of hip-radiographs and DXA images performed on the same day, found concerning inter-method differences in assessing the best-fitting circle around the femoral head [34]. These discrepancies might reflect positional as well as technical differences between the methods. We would argue that this problem also might apply to statistical shape modelling, using landmark points to define subtle differences in shape of the proximal femur.

Overall, confirmed AD was more prevalent in those treated with an abduction device during infancy whilst breech increased the risk of a bilateral AD-tendency. The prevalence of AD and the associations with breech and early abduction treatment found in this study are consistent with previous reports [35]. However, the association found in males between childhood growth patterns and bilateral tendency to acetabular overcoverage, is a novel finding. This contrasts with results from a recent Dutch study demonstrating a negative association of both BMI and physical activity with acetabular dysplasia in 9-year-olds [36]. Although this study included 1188 individuals, DXA images were however only made for the final 20% of the children who visited the research centre. This, in addition to using DXA images and JPEG format for image analysis, might to some extents have biased the results.

Interestingly, young women with a bilateral tendency to acetabular overcoverage were characterized by higher size of height trajectories. No associations were observed with weight in young women. We have no explanations for these findings, which warrant further investigation.

To our knowledge, this is the first study to investigate associations between acetabular shape at skeletal maturity as defined by six different radiological phenotypes, and growth parameters, including multiple height and weight measurements during infancy and childhood. The population-based design of the initial RCT, partly conserved for the follow-up cohort, and the use of inverse probability-weights to take account of the study sampling design and non-responders, strengthen our findings. In addition, the protocol of high-quality hip radiographs was highly standardised, and the radiographs were meticulously analysed to define five different acetabular phenotypes. Another strength of this study is the retrieval of multiple anthropometric measures during childhood, which increases the accuracy of growth trajectory estimates during childhood. SITAR accounts for the nonlinear shape of infant growth and has the advantage of efficiently estimating growth trajectories and using all the available data irrespective of measurement timing or frequency [19]. We acknowledge several limitations to our study. First, only a subgroup of the large, initial RCT cohort was invited for the follow up, and only around 60% of the invited young adults attended follow up. This represents a potential impact of non-random missing data. To mitigate this potential attendance bias, rigorous measures have been taken in all steps of the statistical analyses, including inverse probability-weights to take account of the study sampling design and non-response. Of the 2279 attending follow-up participants, only 1764 (77.4%) had complete growth data available. Furthermore, we chose to include the pincer-type of femoroacetabular impingement, as this represents an overcoverage of the femoral head by the acetabulum. However, we did not include the alpha-angle measurement, representing the cam-type femoroacetabular impingement, as it evaluates the femoral head-neck junction, and not the shape of the acetabulum.

Based on a robust, longitudinal design, this study suggests an association between growth patterns during infancy and childhood, and acetabular shape as assessed on radiographs at skeletal maturity. For males, bilateral tendency to acetabular overcoverage was associated with higher weight velocity in childhood, and bilateral tendency to acetabular overcoverage and was associated with tempo of BMI in childhood. These associations give insight into the importance of prepubertal growth for future hip-health and osteoarthrosis risk. Future research might elaborate on these findings.

## Conclusion

Our findings suggest that individual growth patterns in childhood are associated with modest variations in acetabular shape at skeletal maturity.

## Supplementary Information

Supplementary Material 1.

## Data Availability

De-identified individual participant data will be made available. The data will be made available upon publication to researchers who provide a methodologically sound proposal for use in achieving the goals of the approved proposal. Proposals should be submitted to karen.rosendahl@unn.no.

## Abbreviations

AD: Acetabular dysplasia
ADR: Acetabular depth-width ratio
BMI: Body mass index
CE: Centre-edge angle
DDH: Developmental dysplasia of the hip
FAI: Femoroacetabular impingement
FHEI: Femoral head extrusion index
OA: Osteoarthritis
RCT: Randomized controlled trial
SITAR: SuperImposition by Translation And Rotation
