# Supplementary material for: Childhood growth associated with hip shapes at skeletal maturity: the Bergen Hip Cohort Study
Source: BMC Musculoskelet Disord. 2025 Dec 30;27:86. doi: 10.1186/s12891-025-09461-7 (PMC12860115; doi:10.1186/s12891-025-09461-7)
Supplement: Supplementary file 1 — Supplementary Material 1. [file 12891_2025_9461_MOESM1_ESM.docx]

### Suppl. Table S1

Cut off values used to categorize acetabular shape parameters, based on 841 males and 1170 females at 18-19yrs of age, from the original Bergen Hip Cohort.

|  | **Normal** | | **Borderline AD** | **AD** | **Borderline**  **FAI** | **FAI** |
| --- | --- | --- | --- | --- | --- | --- |
| **Right hip** | | | | | | |
| Sharp angle  -male  -female | | < 42.3°  < 44.2° | (42.3°; 45.8°)  (44.2°; 47.7°) | > 45.8°  > 47.7° |  |  |
| Acetabular depth ratio  -male  -female | | > 259.6‰  > 261.9‰ | (224.7‰; 259.6‰]  (226.1‰; 261.9‰] | < 224.7‰  < 226.1‰ |  |  |
| Wiberg angle  -male  -female | | (26.0°; 38.2°]  (24.0°; 36.2°] | (19.9°; 26.0°]  (17.9°; 24.0°] | < 19.9°  < 17.9° | [38.2°; 45.0°)  [36.2°; 43.4°) | > 45.0⁰  > 43.4° |
| Femoral head extrusion index  -male  -female | | [79.3%; 91.9%)  [79.0%; 92.2%) | [73.0%; 79.3%)  [72.4%; 79.0%) | < 73.0%  < 72.4% | [91.9%; 98.2%)  [92.2%; 98.8%) | > 98.2%  > 98.8% |
| **Left hip** | | | | | | |
| Sharp angle  -male  -female | | < 42.2°  < 44.4° | (42.2°; 45.7°)  (44.4°; 47.3°) | > 45.7°  > 48.0° |  |  |
| Acetabular depth ratio  -male  -female | | > 265.0‰  > 264.7‰ | (232.8‰; 265.0‰]  (229.4‰; 264.7‰] | < 232.8‰  < 229.4‰ |  |  |
| Wiberg angle  -male  -female | | (27.0°; 38.6°]  (25.4°; 37.4°] | (21.2°; 27.0°]  (19.4°; 25.4°] | < 21.2°  < 19.4° | [38.6°; 44.4°)  [37.4°; 43.4°) | > 44.4⁰  > 43.4° |
| Femoral head extrusion index  -male  -female | | [80.9%; 92.9%)  [80.1%; 93.5%) | [74.9%; 80.9%)  [73.4%; 80.1%) | < 74.9%  < 73.4% | [92.9%; 98.9%)  [93.5%; 100.2%) | > 98.9%  > 100.2% |

### Suppl. Table S2 Prevalence of the measured acetabular shape categories within the four latent classes (Normal, Tendency AD, AD and Acetabulum overcoverage). Left and Right hip.

| **Left hip** |  | **Normal** | | | **Tendency AD** | | | | **AD** | | | | **Acetabular Overcoverage** | | | |
| --- | --- | --- | --- | --- | --- | --- | --- | --- | --- | --- | --- | --- | --- | --- | --- | --- |
|  |  | **1,214** | **68.8** | | **262** | | **14.9** | | **32** | | **1.8** | | **256** | | **14.5** | |
|  |  | n | % | | n | | % | | n | | % | | n | | % | |
| **Sharp's angle** | **Normal** | 1,081 | 89.0 | | 158 | | 60.3 | | 7 | | 21.9 | | 256 | | 100.0 | |
|  | **Borderline AD** | 121 | 10.0 | | 78 | | 29.8 | | 13 | | 40.6 | | 0 | | 0.0 | |
|  | **AD** | 12 | 1.0 | | 26 | | 9.9 | | 12 | | 37.5 | | 0 | | 0.0 | |
|  |  |  |  | |  | |  | |  | |  | |  | |  | |
| **Acetabular depth ratio (ADR)** | **Normal** | 1,113 | 91.7 | | 121 | | 46.2 | | 7 | | 21.9 | | 254 | | 99.2 | |
|  | **Borderline AD** | 95 | 7.8 | | 120 | | 45.8 | | 23 | | 71.9 | | 2 | | 0.8 | |
|  | **AD** | 6 | 0.5 | | 21 | | 8.0 | | 2 | | 6.3 | | 0 | | 0.0 | |
|  |  |  |  | |  | |  | |  | |  | |  | |  | |
| **Centre-edge (CE) angle of Wiberg** | **Normal** | 1,210 | 99.7 | | 22 | | 8.4 | | 0 | | 0.0 | | 0 | | 0.0 | |
|  | **Borderline AD** | 0 | 0.0 | | 240 | | 91.6 | | 0 | | 0.0 | | 0 | | 0.0 | |
|  | **AD** | 0 | 0.0 | | 0 | | 0.0 | | 32 | | 100.0 | | 0 | | 0.0 | |
|  | **Borderline FAI** | 4 | 0.3 | | 0 | | 0.0 | | 0 | | 0.0 | | 211 | | 82.4 | |
|  | **FAI** | 0 | 0.0 | | 0 | | 0.0 | | 0 | | 0.0 | | 45 | | 17.6 | |
|  |  |  |  | |  | |  | |  | |  | |  | |  | |
| **Femoral head extrusion index (FHEI)** | **Normal** | 1071 | 88.2 | | 71 | | 27.1 | | 0 | | 0.0 | | 157 | | 61.3 | |
|  | **Borderline AD** | 51 | 4.2 | | 180 | | 68.7 | | 8 | | 25.0 | | 0 | | 0.0 | |
|  | **AD** | 2 | 0.2 | | 10 | | 3.8 | | 24 | | 75.0 | | 0 | | 0.0 | |
|  | **Borderline FAI** | 81 | 6.67 | | 1 | | 0.38 | | 0 | | 0 | | 60 | | 23.44 | |
|  | **FAI** | 9 | 0.7 | | 0 | | 0.0 | | 0 | | 0.0 | | 39 | | 15.2 | |
| **right hip** |  | **Normal** | | | | **Tendency AD** | | | | **AD** | | | | **Acetabular Overcoverage** | | |
|  |  | **1,172** | | **66.4** | | **246** | | **13.9** | | **39** | | **2.2** | | **307** | | **17.4** |
|  |  | **n** | | **%** | | **n** | | **%** | | **n** | | **%** | | **n** | | **%** |
| **Sharp's angle** | **Normal** | 1,049 | | 89.5 | | 126 | | 51.2 | | 4 | | 10.3 | | 306 | | 99.7 |
|  | **Borderline AD** | 108 | | 9.2 | | 108 | | 43.9 | | 23 | | 59.0 | | 1 | | 0.3 |
|  | **AD** | 15 | | 1.3 | | 12 | | 4.9 | | 12 | | 30.8 | | 0 | | 0.0 |
|  |  |  | |  | |  | |  | |  | |  | |  | |  |
| **Acetabular depth ratio (ADR)** | **Normal** | 1,064 | | 90.8 | | 133 | | 54.1 | | 16 | | 41.0 | | 306 | | 99.7 |
|  | **Borderline AD** | 106 | | 9.0 | | 95 | | 38.6 | | 16 | | 41.0 | | 1 | | 0.3 |
|  | **AD** | 2 | | 0.2 | | 18 | | 7.3 | | 7 | | 18.0 | | 0 | | 0.0 |
|  |  |  | |  | |  | |  | |  | |  | |  | |  |
| **Centre-edge (CE) angle of Wiberg** | **Normal** | 1,170 | | 99.8 | | 42 | | 17.1 | | 0 | | 0.0 | | 10 | | 3.3 |
|  | **Borderline AD** | 0 | | 0.0 | | 204 | | 82.9 | | 10 | | 25.6 | | 0 | | 0.0 |
|  | **AD** | 0 | | 0.0 | | 0 | | 0.0 | | 29 | | 74.4 | | 0 | | 0.0 |
|  | **Borderline FAI** | 2 | | 0.2 | | 0 | | 0.0 | | 0 | | 0.0 | | 249 | | 81.1 |
|  | **FAI** | 0 | | 0.0 | | 0 | | 0.0 | | 0 | | 0.0 | | 48 | | 15.6 |
|  |  |  | |  | |  | |  | |  | |  | |  | |  |
| **Femoral head extrusion index (FHEI)** | **Normal** | 1071 | | 91.4 | | 51 | | 20.7 | | 0 | | 0.0 | | 115 | | 37.5 |
|  | **Borderline AD** | 34 | | 2.9 | | 195 | | 79.3 | | 7 | | 18.0 | | 0 | | 0.0 |
|  | **AD** | 1 | | 0.1 | | 0 | | 0.0 | | 32 | | 82.1 | | 0 | | 0.0 |
|  | **Borderline FAI** | 66 | | 5.63 | | 0 | | 0 | | 0 | | 0 | | 133 | | 43.32 |
|  | **FAI** | 0 | | 0.0 | | 0 | | 0.0 | | 0 | | 0.0 | | 59 | | 19.2 |

### Suppl. Table S3

Distribution of the latent acetabular phenotypes in left and right hip and combination in the subject phenotype.

|  |  | **Right hip** | | | | |
| --- | --- | --- | --- | --- | --- | --- |
|  |  | Normal | Tendency to Acetabular Dysplasia | Acetabular Dysplasia | Acetabular Over-coverage | Total (%) |
| **Left hip** | Normal | 910 | 132 | 13 | 159 | 1214  (68.8%) |
|  | Tendency to Acetabular Dysplasia | 148 | 96 | 16 | 2 | 262  (14.9%) |
|  | Acetabular Dysplasia | 4 | 18 | 10 | 0 | 32  (1.8%) |
|  | Acetbular Over-coverage | 110 | 0 | 0 | 146 | 256  (14.5%) |
|  | Total | 1172  (66.4%) | 246  (13.9%) | 39  (2.2%) | 307  (17.4%) | 1764 |

### Suppl. Table S4

### Latent acetabular phenotypes for left and right hip combined to assign each participant a phenotype based on distribution in Table S3

| **Left Hip** | **Right Hip** | **Assigned phenotype** | **Number with assigned phenotype** |
| --- | --- | --- | --- |
| Normal | Normal | Normal | 910 |
| Tendency to Acetabular Dysplasia | Normal | Unilateral tendency to Acetabular Dysplasia | 148 |
| Normal | Tendency to Acetabular Dysplasia | Unilateral tendency to Acetabular Dysplasia | 132 |
| Tendency to Acetabular Dysplasia | Tendency to Acetabular Dysplasia | Bilateral tendency to Acetabular Dysplasia | 96 |
| Acetabular Dysplasia | Acetabular Dysplasia | Acetabular Dysplasia | 61  *(13+16+10+18+4)* |
| Acatabular over-coverage | Normal | Unilateral Acetabluar Overcoverage | 110 |
| Normal or Tendency to Acetabular Dysplasia | Acetabular overcoverage | Unilateral Acetabluar Over-coverage | 161  (159+2) |
| Acetabular Overcoverage | Acetabular Overcoverage | Acetabular Overcoverage | 146 |

### Suppl. Table S5

Analysis of which variables were found to be the main determinants of attendance at follow up

|  |  | **Attended at follow up** | | | |  |
| --- | --- | --- | --- | --- | --- | --- |
|  | **Invited at Follow up** | **No** | | **Yes** | | **Chi-square test** |
|  | **n = 4297** | **n =2018** | | **n = 2279** | |  |
| **Variable** | **n** | **n** | **%** | **n** | **%** | **p value** |
| **Sex** |  |  |  |  |  |  |
| Male | 2,104 | 1,214 | 60.2 | 890 | 39.1 |  |
| Female | 2,193 | 804 | 39.8 | 1,389 | 60.9 | <0.0001 |
| **Year of Birth** |  |  |  |  |  |  |
| 1988 | 290 | 108 | 5.4 | 182 | 8.0 |  |
| 1989 | 3,821 | 1,837 | 91.0 | 1,984 | 87.1 |  |
| 1990 | 186 | 73 | 3.6 | 113 | 5.0 | <0.0001 |
| **Breech position** |  |  |  |  |  |  |
| No | 4,100 | 1,937 | 96.0 | 2,163 | 94.9 |  |
| Yes | 197 | 81 | 4.0 | 116 | 5.1 | 0.092 |
| **Caesarean section** |  |  |  |  |  |  |
| No | 3,874 | 1,828 | 90.6 | 2,046 | 89.8 |  |
| Yes | 423 | 190 | 9.4 | 233 | 10.2 | 0.375 |
| **Forceps use** |  |  |  |  |  |  |
| No | 4,115 | 1,926 | 95.4 | 2,189 | 96.1 |  |
| yes | 182 | 92 | 4.6 | 90 | 3.9 | 0.322 |
| **Randomization group** |  |  |  |  |  |  |
| General screening | 1,367 | 601 | 29.8 | 766 | 33.6 |  |
| Selective screening | 1,394 | 675 | 33.4 | 719 | 31.5 |  |
| Clinical screening | 1,536 | 742 | 36.8 | 794 | 34.8 | 0.027 |
| **Newborn Ultrasound** |  |  |  |  |  |  |
| No | 2,611 | 1288 | 63.8 | 1,323 | 58.1 |  |
| Yes | 1,686 | 730 | 36.2 | 956 | 41.9 | <0.0001 |
| **Pillow treatment** |  |  |  |  |  |  |
| No | 4,021 | 1,935 | 96.9 | 2,066 | 90.7 |  |
| Yes | 276 | 63 | 3.1 | 213 | 9.3 | <0.0001 |
|  |  |  |  |  |  | **t test** |
| **Birth weight (g)** | **Mean** | **Mean** | **SD** | **Mean** | **SD** | **p value** |
| Male | 3,617 | 3,604.3 | 522.7 | 3,634.1 | 541.3 | 0.204 |
| Female | 3,497 | 3,490.3 | 480.5 | 3,500.8 | 486.5 | 0.627 |

Supplementary information on the Clinical and Radiological examinations at follow up:

The clinical follow-up examination was performed by one of five physicians who were masked to the questionnaires or radiological findings. One experienced senior orthopaedic surgeon standardised the clinical examination and trained the other four. A standardised protocol was developed comprising measurement of body weight and standing height; assessment of joint laxity based on scored evaluations of elbow, knee, and first and fifth finger hypermobility, and the ability to touch the floor with the palm of the hands standing with straight legs to construct a Beighton score (1). A Beighton score ≥4 indicates joint hypermobility. Hip flexion, abduction and adduction were measured with the subject supine, while extension, internal and external rotation were measured prone with 90 degrees flexion of the knee joint. Standing height was measured in cm to the nearest 0.1 cm using a wall-mounted stadiometer and body weight in kg to the nearest 0.1kg using digital scales. Each participant was measured twice by the same observer and the average of the two measurements taken.

Participants were sent a questionnaire to complete before their follow-up visit eliciting information about hip-related problems and a family history of congenital hip disorders in first degree relatives. A second self-complete questionnaire, administered on arrival, obtained information regarding hip pain in the preceding three months, hip problems, and hours per week engaged in moderate and vigorous physical activity outside of school (2).

For the radiographs, all male participants were offered a gonadal shield and female participants were excluded if their pregnancy status was uncertain. Two views were obtained according to a standardised protocol: a weight bearing anteroposterior (AP) view and a supine frog leg view.

1. Beighton P, Horan F. Orthopaedic aspects of the Ehlers-Danlos syndrome. J Bone Joint Surg Br. 1969;51(3):444-53.

2. Bellamy N, Buchanan WW, Goldsmith CH, Campbell J, Stitt LW. Validation study of WOMAC: a health status instrument for measuring clinically important patient relevant outcomes to antirheumatic drug therapy in patients with osteoarthritis of the hip or knee. J Rheumatol. 1988;15(12):1833-40.
